# Quantitative evaluation of thyroid bed accumulation in I-131 radiotherapy: a comparison of laryngeal edema and non-edematous cases

**DOI:** 10.1186/s13550-025-01310-x

**Published:** 2025-09-26

**Authors:** Kenta Konishi, Kohei Wakabayashi, Tomoyuki Asao, Ryo Kokubo, Shuhei Aramaki, Tsutomu Ikenohira, Haruka Fujita, Katsumasa Nakamura

**Affiliations:** 1 Department of Radiation Oncology, Hamamatsu University School of Medicine, 1-20-1, Handayama, Chuo-ku, Hamamatsu, Shizuoka 431-3192 Japan; 2 Division of Radiation Oncology, Shizuoka Cancer Center Hospital, Nagaizumi-cho, Shizuoka 411-8777 Japan

## Introduction

The standard treatment for patients with differentiated thyroid cancer (DTC) is iodine-131 (I-131) radioactive iodine therapy (RAI) after a total thyroidectomy [1]. Radiation sickness and radiation sialadenitis are acute adverse events that have frequently been associated with RAI; they can be treated relatively easily with medications or follow-up observation. Laryngeal edema is a rare acute adverse event after RAI. Although the frequency of this event is low, if laryngeal edema does occur, there is a risk of airway obstruction—a potentially serious complication.

The cause of laryngeal edema has not been established. Two possible causes are radiation-induced inflammation from remnant thyroid tissue and allergy [2]. The application of single photon emission computed tomography (SPECT) has provided accurate absolute quantifications of radioisotopeuptake [3], and new image-analysis software can measure the quantitative parameters including the standardized uptake value (SUV) and the absolute radioactivity concentration of uptake sites. Our research group has reported the quantitative evaluation of thyroid bed uptake in adjuvant radioactive iodine therapy for differentiated thyroid cancer [4]. We have also encountered three patients with post-RAI laryngeal edema whose quantitative uptake values in the thyroid bed were very high compared to cases without edema. Before their I-131 RAI therapy, two of the patients underwent a total thyroidectomy and the third underwent a near-total thyroidectomy. In this study, we compared the patients'quantitative thyroid-bed values with those of similar patients who did not develop laryngeal edema.

## Methods of quantitative evaluation

The patients'I-131 whole-body scan and SPECT/CT were conducted 3 days after the administration of RAI with a SPECT/CT system (Symbia Intevo 6, Siemens Healthcare, Erlangen, Germany). We used Syngo.via imaging software (Siemens Healthcare), which allowed us to measure the SUV and absolute radioactivity concentration (kBq/mL) in the lesion with the highest uptake. Volume of interest (VOI) segmentation was performed based on 40% of the maximum SUV value, and quantitative parameters (SUV and kBq/mL) were then calculated within the defined VOI This fixed-percentage threshold segmentation is an inbuilt contouring method recommended by Siemens, in which all voxels within a user-defined sphere with values > 40% of the maximum voxel value are included to determine the region of interest (ROI). The maximum SUV (SUVmax), mean SUV (SUVmean), maximum kBq/mL (max kBq/mL), and mean kBq/mL (mean kBq/mL) within the defined ROI were automatically calculated. The method was carried out as described [4].

We compared the SUV and kBq/mL values of the thyroid bed of the present three patients with those of 14 patients identified in our previous study [4] who did not develop laryngeal edema after a total thyroidectomy and the administration of 3.70 GBq of I-131.

## Case reports

### Patient 1

A 61-year-old Japanese woman had undergone a right thyroid lobectomy for papillary carcinoma of the thyroid 20 years ago. Eight years later, she underwent a right cervical resection due to a recurrence at cervical lymph nodes. Three years later, a right paratracheal lymphadenectomy was performed to remove the tissue surrounding the right lobe of the thyroid gland. Seven years after that, cervical lymph-node and lung metastases appeared, and a dissection of the remaining thyroid and neck metastases was performed.

**Fig. 1:**
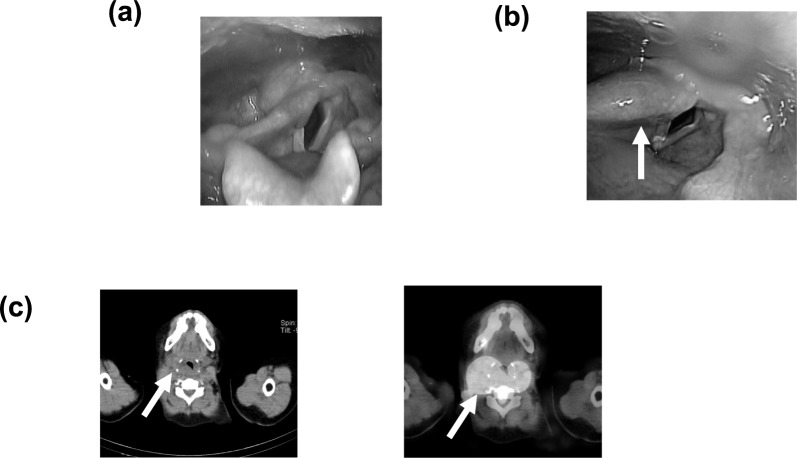
Patient 1, 61-year-old woman. **a** Laryngoscopy before the patient's radioactive iodine therapy (RAI). **b** Laryngoscopy performed 3 days after the same patient's RAI. Edema was observed extending from the right hypopharynx to the arytenoid region. **c** SPECT/CT performed 3 days after RAI. A CT scan revealed swelling in the retropharyngeal space and airway narrowing. A SPECT/CT scan demonstrated intense uptake in the thyroid bed

Three months after that surgery, the patient underwent an iodine-controlled diet for 2 weeks and thyroid-hormone withdrawal, and she was then administered 3.70 GBq of I-131. Approximately 30 h after the administration, she experienced the sensation of tightness in the neck and respiratory distress. However, the symptoms were mild, she did not require any treatment, and she was monitored. Two days later, laryngoscopy was performed because there was no improvement in symptoms. The laryngoscopy demonstrated that the patient's epiglottis was swollen, the posterior interpharyngeal space had narrowed, and the airway was constricted (Fig. 1). The patient's symptoms gradually improved with careful observation alone, and she was discharged 4 days after the RAI administration as planned. A SPECT/CT examination demonstrated a very high uptake of I-131 in the patient's thyroid bed.

### Patient 2

This 54-year-old Japanese woman had undergone a subtotal thyroidectomy and right neck dissection for poorly differentiated carcinoma of the thyroid 20 months ago. The pathological stage was T1bN1bM0, and 5 of the 12 lymph nodes were positive. Ultrasonography revealed approximately 4 mL of thyroid tissue remaining, but performing a reoperation was considered difficult due to the risk of recurrent laryngeal nerve palsy.

Five months later, the patient was treated with 3.70 GBq I-131 for the removal of the remnant tissue and as adjuvant therapy. Before the treatment, a 2-week iodine-controlled diet and thyroid hormone withdrawal were performed.

**Fig. 2:**
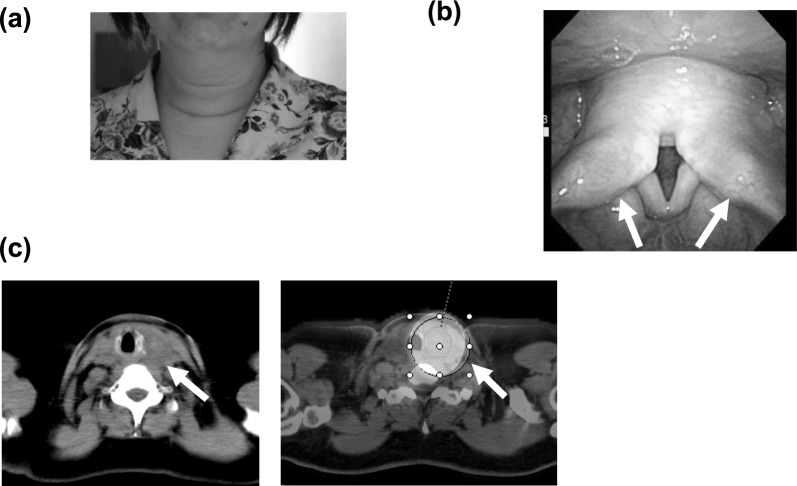
Patient 2, a 54-year-old woman. **a** Visual inspection 2 days after RAI confirmed swelling in the neck. **b** Laryngoscopy performed 3 days after RAI revealed edema in the bilateral arytenoid regions and vocal cords. **c** A CT scan shows mild edematous appearance of the residual thyroid tissue. A SPECT/CT scan demonstrated intense uptake in the residual thyroid tissue

Twenty-four hours after the RAI administration, the patient started to experience neck swelling and dyspnea. Laryngoscopy revealed edema of the bilateral larynx and vocal cords (Fig. 2). 10 mg of oral prednisolone was administered, and at 1 week later the patient's symptoms had improved and the prednisolone was discontinued. SPECT/CT showed strong I-131 uptake in the neck.

### Patient 3

**Fig. 3:**
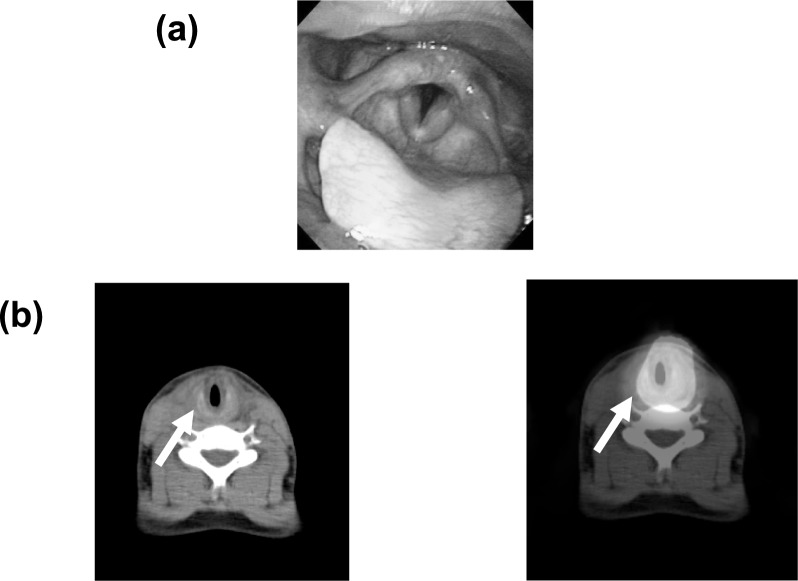
Patient 3, a 29-year-old man. **a** Laryngoscopy performed 2 days after RAI revealed mild edema; however, the vocal cords were adequately open. **b** SPECT/CT revealed mild laryngeal edema but strong uptake in the thyroid bed

A 29-year-old Japanese man had undergone a left thyroid lobectomy and left selective neck dissection for papillary adenocarcinoma 11 years earlier. The pathological stage was unknown. Ten years later, he underwent a completion thyroidectomy and neck dissection due to a recurrence of cervical lymph nodes. He also had multiple lung metastases. Three months after the surgery, he received a 2-week iodine-controlled diet and thyroid-hormone withdrawal and was then administered 3.70 GBq of I-131. Two days after this therapy, he experienced dysphonia and dysphagia. Laryngoscopy revealed slight edema of the vocal cords (Fig. 3). Although the glottis was adequately open, antiallergic medication was initiated as a precaution. SPECT/CT showed strong I-131 uptake in the neck.

## Results

Three patients developed laryngeal edema after receiving 3.70 GBq of I-131. All showed strong I-131 uptake in the thyroid bed on SPECT/CT. Compared to 14 patients without edema, these three patients had significantly higher SUVmax, SUVmean, maximum kBq/mL, and mean kBq/mL values (Table 1). This suggests a possible association between high radiation dose to the thyroid bed and the occurrence of laryngeal edema. Among the three, Patient 2 showed markedly higher quantitative values, which may be attributable to the larger volume of remnant thyroid tissue (approximately 4 mL) remaining after subtotal thyroidectomy.

**Table 1:** Comparison of quantitative values in cases with and without laryngeal edema

	Patient 1	Patient 2	Patient 3	Study [4] n = 14	*p* value*
SUVmax	43.2	337.04	75.5	4.52 ± 3.54	< 0.01
SUVmean	25	242.34	30.2	2.70 ± 2.11	< 0.01
maximum kBq/mL	2608	15,083	2909	196.4 ± 143.8	< 0.01
mean kBq/mL	1510	10,845	1163	117.3 ± 85.7	< 0.01

The quantitative values in the cases with laryngeal edema were significantly higher than those of the 14 previous cases without laryngeal edema

*Mann–Whitney U test

## Discussion

At our institution, approximately 70 patients are treated with I-131 therapy at a standard dose of 3.70 GBq each year, and from the beginning of quantitative measurements in 2017 to the completion of this report in mid-2025, we have encountered only three patients with laryngeal edema. The occurrence of laryngeal edema following radioactive iodine therapy is rare, with only a small number of cases documented. However, Kinuya et al. reported that three (2.9%) of 102 patients who underwent I-131 therapy for thyroid cancer developed laryngeal edema, suggesting that the incidence of laryngeal edema might be higher than previously thought [5]. Particularly in Japan, when doses of 1.11 GBq or more are administered, patients must be hospitalized in treatment rooms, requiring isolation for a certain period. If laryngeal edema occurs during this isolation period, it could lead to significant confusion regarding the treatment site.

Bal et al. reported that 15 (16.1%) of 93 patients who received 0.55–2.22 GBq of I-131 after undergoing a hemithyroidectomy for thyroid cancer developed laryngeal edema [6]. Among them, three patients were treated with prednisolone; the remaining patients were managed with analgesics alone. Bal et al. noted that they considered radiation-induced thyroiditis to be the cause of laryngeal edema in each case, but their report does not mention differences in quantitative values of the thyroid bed between the cases with and without edema.

Kinuya et al. suggested that the mechanism of laryngeal edema caused by RAI remains largely unclear but the edema is often improved with antiallergic medications and hydrocortisone, leading them to speculate that some form of allergic reaction might be involved [7]. Similarly, Goolden et al. suggested that an allergic reaction to degenerated thyroid tissue might be a potential mechanism for dyspnea occurring within 48 h after I-131 administration [2].

Although our present sample size is limited to the cases of three patients with laryngeal edema, all three patients demonstrated significantly higher quantitative values in the thyroid bed compared to 14 patients without edema. This suggests that high I-131 uptake in the thyroid bed might be a contributing factor to the development of laryngeal edema.

Although not directly related to the study’s primary aim, we note that external radiation exposure at a distance of 1 m from the patients was measured 48 h after I-131 administration as part of routine discharge criteria in Japan (< 30 μSv/h). However, no consistent relationship between this measurement and the occurrence of laryngeal edema was observed in the present cases.

Landström et al. suggested, based on their own cases and a review of 4studies, that residual thyroid tissue and high doses of I-131 might contribute to the development of laryngeal edema [8]. However, they also did not mention differences in the quantitative values of the thyroid bed uptake between cases with and without edema. In contrast, our study provides novel findings by quantitatively demonstrating that the radiation dose to the thyroid bed in cases with laryngeal edema was significantly higher compared to cases without edema.

One limitation of this study is the small sample size (n = 3). A further accumulation of cases is necessary to validate these findings.

## Conclusions

In summary, we quantitatively confirmed that I-131 accumulated to a high degree in patients who developed laryngeal edema, and our findings suggest that an extremely high I-131 uptake in the thyroid bed could be one of the causes of laryngeal edema.

## Data Availability

The datasets used and/or analysed during the current study are available from the corresponding author on reasonable request.

## Abbreviations

DTC: Differentiated thyroid cancer)
RAI: Radioactive iodine therapy
SPECT: Single photon emission computed tomography
SUV: Standardized uptake value
CT: Computed tomography
VOI: Volume of interest
ROI: Region of interest
